# Editorial: Case reports in skin cancer: 2025

**DOI:** 10.3389/fonc.2026.1964103

**Published:** 2026-08-25

**Authors:** Gianluca Nazzaro, Elisa Zavattaro

**Affiliations:** 1 Department of Physiopatology and Transplantation, Università degli Studi di Milano; Dermatology Unit, Fondazione IRCCS Ca’ Granda Ospedale Maggiore Policlinico, Milan, Italy; 2 Department of Health Sciences, Università del Piemonte Orientale Amedeo Avogadro, Dermatology Unit, University Hospital Maggiore della Carità, Novara, Italy

**Keywords:** case report, dermatology, lymphoma, melanoma, non melanoma skin cancer, skin cancers, skin tumor

## Beyond rarity: how case reports continue to shape skin cancer management

The management of skin cancer has undergone a profound transformation over the past decade. Advances in molecular diagnostics, targeted therapies, immune checkpoint inhibitors, and multidisciplinary care have expanded the therapeutic possibilities available to patients while, at the same time, increasing the complexity of clinical decision-making. Clinicians are increasingly confronted with uncommon tumor subtypes, atypical clinical presentations, unexpected treatment responses, and novel therapy-related adverse events that may fall outside the scope of established guidelines or clinical trial evidence.

In this evolving landscape, case reports continue to occupy a unique and valuable place within the medical literature. Although they cannot replace the evidence generated by randomized clinical trials or large observational studies, carefully documented clinical observations may represent the first signal of an emerging phenomenon, an unexpected association, or a potentially innovative therapeutic strategy. This is particularly relevant in dermato-oncology, where many malignancies are rare and where the heterogeneity of clinical presentations and treatment responses frequently limits the feasibility of large prospective studies.

The articles collected in the Research Topic *Case Reports in Skin Cancer: 2025* illustrate this enduring value. Together, they portray contemporary dermato-oncology as a field in which clinical observation, diagnostic reasoning, therapeutic personalization, and multidisciplinary collaboration remain central to patient care. Rather than simply documenting unusual diseases, these reports highlight a broader concept: in skin cancer, the exception may often be the starting point for a new clinical question. Herein, we report the Research Topic published articles as grouped in categories, according to their common denominator.

## When the diagnosis is not what it seems

Accurate diagnosis remains the foundation of effective cancer management, yet some of the most challenging cases arise precisely when clinical, dermoscopic, histopathological, and radiological findings do not immediately point toward a single diagnosis.

Several contributions in this Research Topic illustrate the diagnostic complexity of cutaneous malignancies. The report describing a completely regressed cutaneous melanoma revealed only by the subsequent detection of lymph node metastasis is a particularly striking reminder that the absence of a visible primary lesion does not necessarily exclude a cutaneous origin.(Wang et al.) Similarly, the case of cutaneous metastatic mucin-producing prostate adenocarcinoma emphasizes the importance of considering metastatic disease in the differential diagnosis of an unusual cutaneous lesion, particularly when its clinical and histopathological features may mimic a primary skin neoplasm.(Yildrim et al.)

Other reports underscore the challenges posed by rare primary cutaneous tumors. Primary cutaneous follicle center lymphoma involving the nasal ala [Han et al.) and primary cutaneous apocrine carcinoma mimicking breast carcinoma (Khosh et al.) demonstrate how uncommon anatomic locations and morphologic similarities can complicate the diagnostic process. In such situations, the integration of clinical examination, dermoscopy, histopathology, and immunohistochemistry is not merely complementary but essential.

These cases remind us that diagnostic uncertainty is not simply an obstacle to overcome. It is often the point at which clinical reasoning becomes most important. In an era of increasingly sophisticated diagnostic technologies, the ability to recognize when a presentation does not fit a familiar pattern remains an essential clinical skill.

## Beyond established algorithms: therapeutic innovation in individual patients

If diagnosis represents the first challenge, treatment may be the second. The growing availability of targeted and immune-based therapies has profoundly changed the therapeutic landscape of skin cancer, but it has also raised new questions regarding treatment sequencing, patient selection, and the management of uncommon malignancies.

The report addressing the role of BRAF mutation in the adjuvant and neoadjuvant treatment of melanoma (Di Pietro et al.) exemplifies the increasingly personalized nature of therapeutic decisions. Molecular characteristics are no longer considered merely prognostic or descriptive: they may directly influence the timing and selection of systemic therapy and the overall treatment strategy.

Similarly, the use of programmed cell death protein 1 (PD-1) monoclonal antibodies in rare cutaneous malignancies illustrates how therapeutic principles developed in more common cancers may potentially be extended to diseases for which prospective evidence remains limited (Falk et al). The clinical experience described in these cases cannot establish universal treatment recommendations, but it may provide an important rationale for future investigation and contribute to the growing body of real-world evidence supporting individualized treatment decisions.

The therapeutic innovation presented in this Research Topic is not limited to systemic treatment. The use of talimogene laherparepvec in mucosal melanoma (Ingason et al.) highlights the potential role of oncolytic immunotherapy in a particularly challenging setting, while the combination of modified surgery and photodynamic therapy for basal cell carcinoma in special facial regions (Fan et al.) illustrates how complementary treatment modalities may be integrated to address both oncologic control and the practical challenges associated with anatomically complex sites.

These experiences share a common message: clinical guidelines provide essential frameworks, but the individual patient does not always fit neatly within an algorithm. Personalized medicine therefore requires not only access to new treatments, but also the clinical judgment necessary to adapt established strategies to exceptional circumstances.

## Rare cancers: where individual experience becomes collective knowledge

Rare cutaneous malignancies represent a particularly important setting for case-based evidence. Because of their low incidence and biological heterogeneity, many of these tumors are unlikely to be evaluated through large randomized clinical trials. In such contexts, individual clinical experiences may represent an important source of information regarding natural history, diagnostic pitfalls, and potential therapeutic approaches.

The cases of eccrine porocarcinoma (Yang et al.), primary cutaneous adenoid cystic carcinoma (Geng et al.), and primary cutaneous apocrine carcinoma (Khosh et al.) included in this Research Topic exemplify this challenge. The report of eccrine porocarcinoma arising after minor trauma in a patient receiving long-term carbamazepine further illustrates how apparently incidental clinical circumstances may raise questions about potential contributing factors and mechanisms,(Yang et al.) while the case of adenoid cystic carcinoma with multiple metastases emphasizes the complexity of managing rare tumors once they enter an advanced stage.(Geng et al.)

The value of these reports lies not necessarily in providing definitive answers, but in making exceptional clinical experiences visible. A single case cannot define a standard of care, but it can generate a hypothesis, identify a potential therapeutic signal, or alert clinicians to a previously underrecognized presentation. When multiple observations accumulate over time, these individual experiences may ultimately become the foundation for larger studies and more structured clinical evidence.

## Treating the patient while preserving the possibility of effective cancer therapy

The development of immune checkpoint inhibitors has introduced another important dimension to contemporary dermato-oncology: the management of treatment-related adverse events.

The cases of recurrent supraglottitis associated with combined ipilimumab and nivolumab therapy (Palmeri et al.) and immune checkpoint inhibitor-induced bullous pemphigoid (Grüninger et al.) illustrate the complexity of balancing oncologic efficacy with immune-mediated toxicity. These complications may affect organs well beyond the tumor itself and can become a critical determinant of whether an effective anticancer treatment can be continued.

Particularly illustrative is the experience of using dupilumab to manage checkpoint inhibitor-induced bullous pemphigoid in a patient with progressive melanoma, allowing the initiation or continuation of dual immunotherapy.(Grüninger et al.) Such cases highlight a conceptual shift in cancer care. The objective is no longer simply to recognize and treat an adverse event; it is to manage toxicity in a way that may preserve access to potentially life-prolonging oncologic treatment.

This increasingly complex interface between oncology and dermatology underscores the importance of multidisciplinary collaboration. Dermatologists are becoming essential partners in the care of patients receiving immunotherapy, not only because of their expertise in cutaneous adverse events but also because timely recognition and targeted management of toxicity may influence the broader oncologic trajectory.

## From the exceptional patient to the next clinical question

Although each contribution to this Research Topic focuses on an individual patient or a limited clinical experience, together these reports reveal recurring themes that extend well beyond the cases themselves: diagnostic uncertainty, rare tumor biology, personalized treatment selection, therapeutic innovation, and the management of increasingly complex treatment-related toxicities.

The common thread is perhaps the importance of observation. Progress in dermato-oncology does not always begin with a clinical trial. Sometimes it begins with a clinician recognizing that a patient does not fit the expected pattern, questioning an established assumption, or attempting a different therapeutic approach when conventional options are limited. The careful documentation and dissemination of that experience allow an observation made in one patient to become accessible to the wider medical community.

In this sense, case reports are far more than educational anecdotes. They are instruments of hypothesis generation and, on occasion, the earliest documentation of developments that may later influence clinical practice. Their contribution is particularly relevant in a field such as skin cancer, where rare diseases, rapidly evolving therapeutic strategies, and complex immune-mediated toxicities frequently intersect.

As precision medicine continues to reshape the management of skin cancer, the ability to recognize, document, and share exceptional clinical experiences will remain an essential component of scientific progress. The cases presented in *Case Reports in Skin Cancer: 2025* remind us that, although each patient may represent an exception, the lessons learned from exceptional patients can ultimately contribute to more accurate diagnoses, more personalized treatments, and better care for many others.

